# Editorial: Infant and child nutrition, physical activity, oxidative stress and inflammatory signaling

**DOI:** 10.3389/fnut.2022.993643

**Published:** 2022-08-23

**Authors:** Jorge Moreno-Fernandez, Julio J. Ochoa, Maria Luisa Ojeda, Javier Díaz-Castro

**Affiliations:** ^1^Department of Physiology, Faculty of Pharmacy, University of Granada, Granada, Spain; ^2^Institute of Nutrition and Food Technology “José Mataix Verdú”, University of Granada, Granada, Spain; ^3^Instituto de Investigación Biosanitaria, Granada, Spain; ^4^Department of Physiology, Faculty of Pharmacy, University of Seville, Seville, Spain

**Keywords:** nutrition, metabolism, physical activity, oxidative stress, inflammatory signaling, public health nutrition

Two key factors for the correct and healthy development of the child are physical activity and diet. During the childhood, participation in physical activity is particularly important and lack of physical activity at this important stage of life can lead to limited cognitive and developmental disorders (1). In general, the practice physical activity has a positive effect on the motor, respiratory, endocrine, cardiovascular, nervous, and immune systems. Also, physical exercise increases blood circulation, which leads to an improvement in the supply of oxygen and nutrients to the brain and stimulates the maturation of the motor regions of the brain, affecting motor development and increasing the behavioral speed of nervous stimuli (2, 3). In addition, physical activity affects the synaptic plasticity and the excitation of neurons that form these synapses (4), improving cognitive abilities, and self-esteem, along with reduced depression rates (5).

In this sense, school-age children who perform vigorous physical activity report much better cognitive functioning, reduced body weight and body mass index, increasing lean mass and reducing fat mass (Diaz-Castro et al.). It has been shown that children who exercise regularly have better biomarkers related to the molecular functionality of adipose tissue and the brain, such as adipsin, adiponectin, osteocrine, lipocalin-2, nerve growth factor, brain derived neurotrophic factor, and irisin among others (Diaz-Castro et al.). Results showing the usefulness of early interventions based on physical activity in children to reduce risk factors related to sedentary lifestyle. Additionally, an adequate level of fitness during childhood seems to protect against metabolic risk, inflammation and oxidative stress decreasing leptin and nerve growth factor levels, contributing to control adiposity at the prepubertal stage (Llorente-Cantarero, Leis et al.).

In short, school-age children who perform vigorous physical activity report much better cognitive functioning and show better and healthy body development. In this sense, prepubertal metabolically healthy obese children are less sedentary, more active, and they have better metabolic profiles than metabolically unhealthy obese subjects (Llorente-Cantarero, Aguilera et al.). However, currently, factors such as modernization, transportation systems or even the wide variety of electronic equipment and electronic devices, have greatly reduced the need for perform physical work, thus encouraging a more sedentary lifestyle. This phenomenon has special attention among children and adolescents, who spend a lot of time using these devices, a fact even promoted in many cases by the family environment (6). For those reasons, the researchers emphasize that, despite these undoubted benefits, only about a third of children play sports regularly (7) and we cannot forget that the lack of physical activity is one of the main causes of childhood obesity and one of the most important public health problems nowadays. Currently, physical inactivity is considered one of the main risk factors for death worldwide, and a risk factor for diseases such as hypertension, dyslipidemia, and diabetes mellitus (8). Lack of physical activity leads to health problems, including postural problems (such as idiopathic scoliosis), somatic conditions, overweight and obesity, circulation problems, and even premature death (9).

Another factor of great importance to consider for the correct development of children is diet. It is clear that diet is related to multiple factors previously mentioned such as neuronal functionality, metabolism, or body composition. It is also, a very important factor in the development of obesity. Diet and exercise have to be factors that must act together, if what is intended is to achieve a healthy development of children (10, 11). In this regard, it has been indicated that the children practicing exercise showed high adherence to the Mediterranean Diet associated with lower incidence of chronic non-communicable diseases, including obesity, and were more concerned about their diet (Diaz-Castro et al.).

In summary, researching the links between diet, exercise, inflammatory stress, oxidative, and growth factors during childhood is important to understand how they contribute to optimal growth and development, deeply influencing health and the results of the above-mentioned studies and reviews represent an enormous amount of new relevant data on the child nutrition, physical activity, oxidative stress and inflammatory signaling. Despite all the existing literature and evidence related to this extremely important topic, the papers published in this e-book clearly show that there are still many aspects to be clarified and understood such as for example, the engagement of physical activity of girls to assess the influence of physical activity on this group, which is more reluctant to do it. After reading this book, some topics as the development of new physical activity protocols designed specifically for school-age children to maximize the benefits of training and minimize the risk of injury, together with the improvement of nutritional knowledge of foods and diets, enabling to acquire the knowledge and facilitating the development of activities development which will contribute to promote health thanks to the implementation of nutritional knowledge and physical activity protocols at school.

## Author contributions

JM-F and JD-C wrote the introduction and the conclusion. JO and MO wrote the central part with comments to the cited papers and references. All authors contributed to the article and approved the submitted version.

## Conflict of interest

The authors declare that the research was conducted in the absence of any commercial or financial relationships that could be construed as a potential conflict of interest.

## Publisher's note

All claims expressed in this article are solely those of the authors and do not necessarily represent those of their affiliated organizations, or those of the publisher, the editors and the reviewers. Any product that may be evaluated in this article, or claim that may be made by its manufacturer, is not guaranteed or endorsed by the publisher.
